# Surgical site infections after stabilization of pelvic ring injuries: a retrospective analysis of risk factors and a meta-analysis of similar studies

**DOI:** 10.1007/s00264-023-05719-8

**Published:** 2023-03-03

**Authors:** Martin Salášek, Richard Český, Adam Whitley, Kryštof Šídlo, Petr Klézl, Valér Džupa

**Affiliations:** 1 Department of Orthopaedics and Traumatology, Faculty of Medicine of Charles University, and University Hospital, Pilsen, Czech Republic; 2 New Technologies for the Information Society, Faculty of Applied Sciences of University of West Bohemia, Alej Svobody 80, 304 60 Plzeň, Czech Republic; 3 Third Faculty of Medicine, Charles University, Prague, Czech Republic; 4 Department of Surgery, Third Faculty of Medicine of Charles University, and University Hospital Kralovske Vinohrady, Prague, Czech Republic; 5 Department of Orthopaedics and Traumatology, Third Faculty of Medicine of Charles University, and University Hospital Kralovske Vinohrady, Prague, Czech Republic; 6 Department of Urology, Third Faculty of Medicine of Charles University, and University Hospital Kralovske Vinohrady, Prague, Czech Republic

**Keywords:** Pelvic ring, Infection, Stabilization, Risk factor analysis, Surgical site infection, Fracture related infection

## Abstract

**Purpose:**

Pelvic ring fractures requiring surgical stabilization are severe injuries. Surgical site infections occurring after stabilization of the pelvis are serious complications, requiring complex and multidisciplinary treatment.

**Methods:**

This is a retrospective observational study from a level I trauma centre. One hundred and ninety-two patients who underwent stabilization of closed pelvic ring injuries without signs of pathological fracture were selected for inclusion into the study. After excluding seven patients for having incomplete data, the final study group consisted of 185 patients (117 men and 68 women). Basic epidemiologic data and potential risk factors were recorded and analyzed by Cox regression, Kaplan–Meier curves, and risk ratios in 2 × 2 tables. Categorical variables were compared by Fisher exact tests and chi squared tests. Parametric variables were analyzed with Kruskal–Wallis tests with post hoc Wilcoxon tests.

**Results:**

Surgical site infections occurred in 13% of the study group (24 from 185). Eighteen infections occurred in men (15.4%) and six in women (8.8%). There were two significant risk factors in women: age over 50 years (*p* = 0.0232) and concomitant urogenital trauma (*p* = 0.0104). The common risk ratio for both these factors was 212.59 (8.78–5148.68), *p* = 0.0010. No significant risk factors were identified in men despite younger men having a higher incidence of infection (*p* = 0.1428).

**Conclusion:**

Overall rate of infectious complications was higher than in the literature, but this might be caused by inclusion of all patients regardless of surgical strategy. Higher age in women and lower age in men were associated with higher infection rates. Concomitant urogenital trauma was a significant risk factor in women.

## Introduction

Pelvic fractures requiring surgical treatment are severe injuries regardless of the patient’s age. They are usually the result of high-energy trauma, such as traffic accidents, falls from height, or impacts from heavy objects. In patients with osteoporosis pelvic fractures can occur following low-energy trauma, such as falls from standing height or less [19].

The stabilization of unstable pelvic ring injuries remains challenging, because of the inadequate posterior soft tissue coverage, complex local anatomy, and biomechanics [11]. Open reduction and internal fixation (ORIF) is recognized as the gold standard for the treatment of unstable posterior pelvic ring injuries. In ORIF, the pelvic and spinopelvic anatomy is reconstructed, and neural elements in the lower lumbar spine and sacrum are decompressed and allow early weight bearing [11]. ORIF is associated with a higher risk of infection in comparison with minimally invasive pelvic ring fixation [22–24]. The surgical treatment of pelvic ring fractures and its complications has been subject to a large number of studies. However, much less research has been devoted to the occurrence of post-operative infectious complications and their risk factors [6, 16]. Furthermore, the majority of studies are focused on complications of only one or two types of implants, which can cause selection bias because of different infection rates in ORIF and minimally invasive fixation. In this study, we therefore chose an unbiased cohort of patients who underwent both treatment approaches. The goal of our study was to evaluate risk factors for surgical site infections after pelvic ring injuries stabilization that required re-operation in treatment management. The indication for reoperation was usually a combination of two or more of the following factors: significant progression of inflammatory parameters, deterioration of wound secretion, clinical signs of possible development of a sepsis despite antibiotics administration, a significant cavity (haematoma) imaged on the ultrasound or CT scan in the area of the surgical wound in case of contraindication to USG or CT assisted puncture and drainage.

## Material and methods

Medical records from a level I trauma centre (University Hospital Kralovske Vinohrady, Prague) between the years 2009 and 2019 were retrospectively screened to identify all patients who underwent operative treatment for pelvic ring injuries. In total, 192 patients were identified during this period. After excluding patients with pathological fractures and patients with incomplete follow-up data, a total of 185 patients were include into the analysis. There was no significant difference in sex ratio when comparing the total number of patients who underwent operative treatment for pelvic ring fractures (119 males and 73 females) with the patients included in the study (117 males and 68 females) (*p* = 0.8318). There were two open pelvic ring injuries, but none was complicated by infection (*p* = 1.0000). Complex pelvic fracture with Morel-Lavallee lesion was revealed in five patients, also in non-infection group (*p* = 1.0000). Superficial skin or subcutaneous tissue infection was revealed in nine patients, and none of them was indicated for revision surgery. These patients were not included in the infection group.

The primary outcome measure was surgical site infection (SSI) requiring operative treatment. During the primary operation, prophylactic cephalosporin antibiotics were administered, and the duration of antibiotic prophylaxis was 24 to 48 h (according to the general risk for development of infectious complications), most often Cefuroxime or Cefazoline. Twenty-four hours duration was used in patients without risky comorbidities, e.g., diabetes, age up 70, chronic immunosuppressive treatment, malnutrition. Longer prophylaxis (> 48 h) was administrated in case of open pelvic ring fractures. During treatment, we used antibiotics according to the recommendations of the microbiological centre—most often Oxacillin and Clindamycin for staphylococcal infections, Ceftriaxone for G-infections, and a combination of crystalline penicillin and Metronidazole for anaerobic infections.

We did not address the issue of orthopaedic surgeon—one experienced surgeon for the operation, in the event that a less experienced one operates, so we considered it irrelevant to evaluate the number of infections according to the surgeon.

The time dependence of cumulative incidence of SSI was graphically expressed using Holt’s linear trend, Sen’s slope, and Mann–Kendall test were used for trend analysis.

Demographic and epidemiological data, including age, sex, and data on the number of pelvic fractures and infectious complications, were collected and analyzed. We then collected and analyzed data concerning the following potential risk factors: mechanism of injury, type of fracture according to the AO/OTA classification, presence of injuries in other body regions, presence of concomitant urogenital trauma, and the type of implants. Injuries were classified according to the injury severity score (ISS) and presence of trauma of other parts of the body as: monotrauma (injuries of only the pelvis with ISS ≤ 15), combined trauma (injuries of the pelvis and other regions, with ISS scores ≤ 15), and polytrauma (multiple trauma, injuries of the pelvis and other regions, with ISS scores ≥ 16).

Due to the disproportionality of the data, we used two models, one for each gender. The significance of the Cox regression model of each group as a whole was evaluated by an LL test approximated to chi-square test. Apart from the Cox regression model, each risk factor was evaluated graphically using a Kaplan–Meier graph and log-rank test. In cases where more than two risk factors were evaluated at once, we used multiple long-rank tests with post hoc comparison using Fisher’s exact test because Bonferroni correction with paired comparisons was too conservative in regard to the distribution of the individual data. The risk ratio for individual risk factors was calculated using 2 × 2 tables with *p*-value approximation using the z-test and also with the in cases with suitable amounts of data from the results of the log-rank test, when the *p*-value was calculated from the chi squared test. The Kaplan–Meier graphs show the time until surgical revision for infectious complications individually for each risk factor.

The groups of women and men were compared using Woolf’s heterogeneity test. Heterogeneity was calculated using common odds ratio (men + women, according to Cochrane-Mantel-Haenzel). In addition to categorical distribution for age (up to 50 and over 50 years), we also evaluated age quantitatively using the Kruskal–Wallis test with post hoc pairwise comparisons using the Mann–Whitney-Wilcoxon exact test, including not only the effect of gender but also the type of injury according to the AO/OTA classification. We considered all *p*-value less than 0.05 as significant. All statistical analyses were performed in MS Excel 2019 (Microsoft, Redmond, WA, USA) using realstat statistical macro (https://www.mdcalc.com/qsofa-quick-sofa-score-sepsis) (https://www.real-statistics.com/). For performing the comparison of the incidence of infections, we calculated the percentage of infections using the equation: *infection* $rate= \frac{n (infection)}{N}$, when *n(infection)* represents the number of infections and *N* represents the total number of patients in the studies. The infection rate was expressed as a percentage from one hundred. The metanalytic like comparison was performed using methods described by Neyeloff et al. 2012 [15].

## Results

From the total cohort of 192 patients, 25 severe infections requiring revision surgery (13.0%, 95% CI 7.9–18.1%) occurred. From the 185 patients included into the study, 24 severe infections occurred (13.0%, 95% CI 7.8–18.2). Eighteen of these infections occurred in the group of males (15.4%) and six in the group of females (8.8%), *p* = 0.2586 (Fisher exact test). The mean time to the first revision surgery (± 1 SD) was 24.3 ± 11.8 days (95% CI: 19.6–29.0), and all re-operations were performed within two months. The average number or re-operations was 1.9 ± 2 (95% CI: 1.0–2.8) in men vs. 1.2 ± 0.4 (95% CI: 0.8–1.5) in women, *p* = 0.6261.

**Fig. 1:**
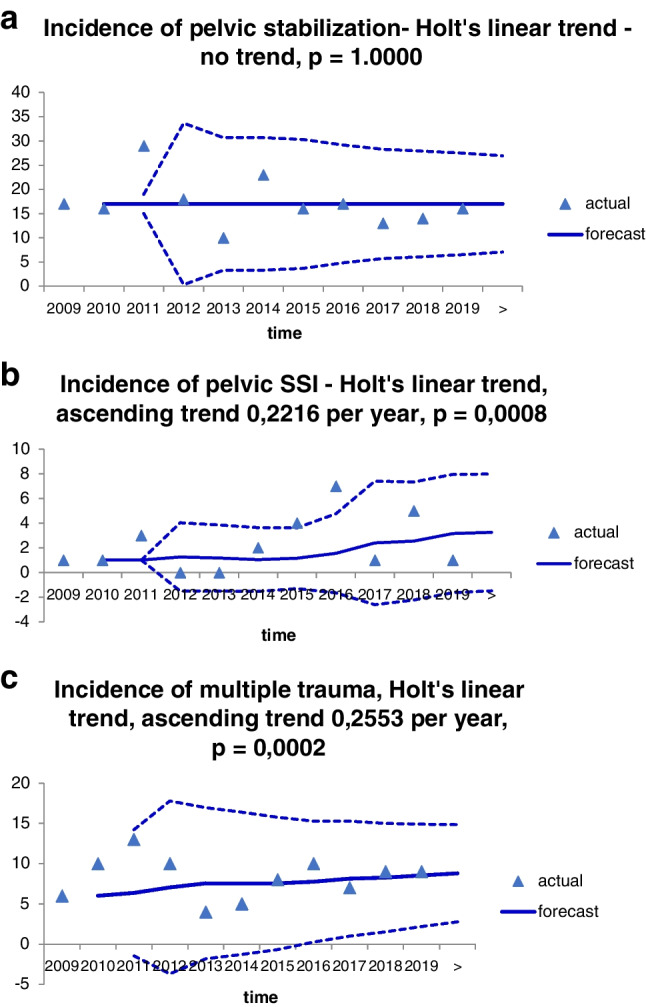
**a** Cumulative incidence of pelvic stabilization. **b** Cumulative incidence of pelvic SSI. **c** Cumulative incidence of multiple trauma

The average annual number of surgical procedures was 17.2 ± 4.8 SD (95% CI: 14.3–20.0). The average annual number of severe infections was 2.3 ± 2.1 SD (95% CI: 1.0–3.5). During the study period, the annual number of infectious complications had ascending trend when using Holt’s linear trend, and this was in concordance with the increasing annual incidence of multiple trauma and in discordance with the total number of pelvic ring stabilization (constant incidence, *p* = 1.0000) (Fig. 1a–c). Sen’s slope for infections was 0.2216 per year (95% CI 0.0579–0.3760, *p* = 0.0008; Sen’s slope for multiple trauma was 0.2553 per year (95% CI 0.1967–0.2924, *p* = 0.0002).

**Fig. 2:**
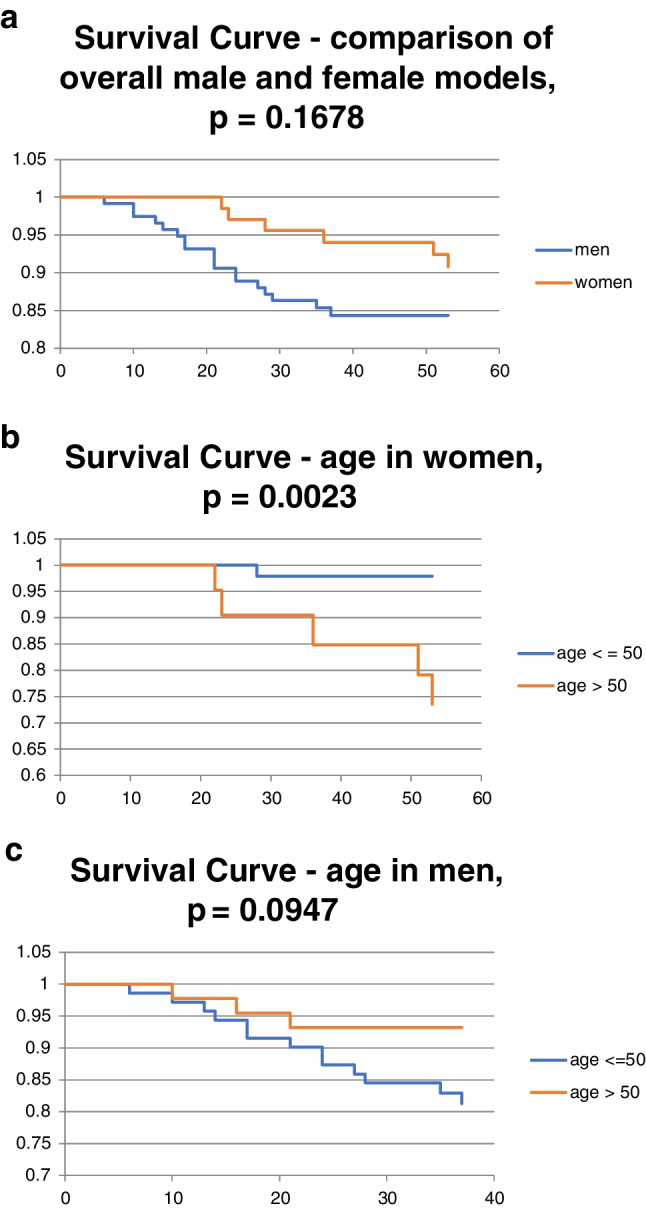
**a** Comparison of overall models. **b** Age in women. **c** Age in men

**Fig. 3:**
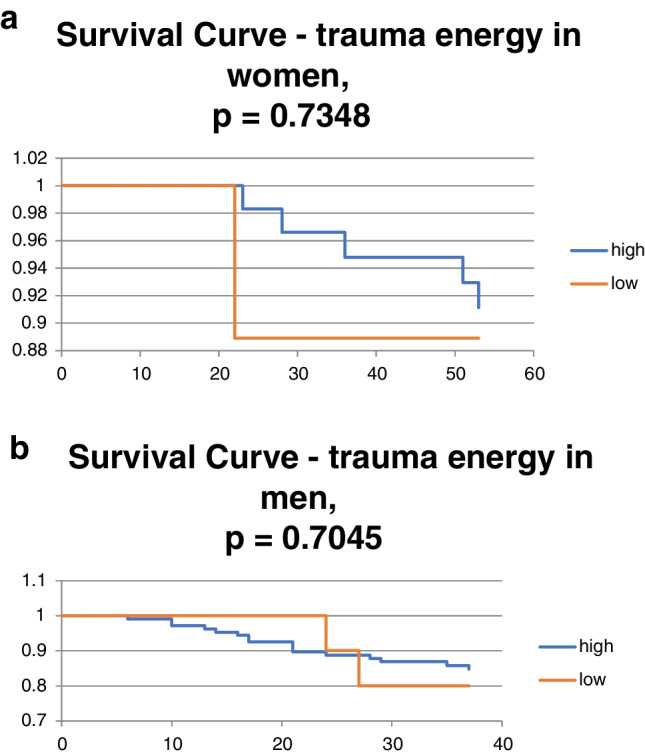
**a** Trauma energy in women. **b** Trauma energy in men

**Fig. 4:**
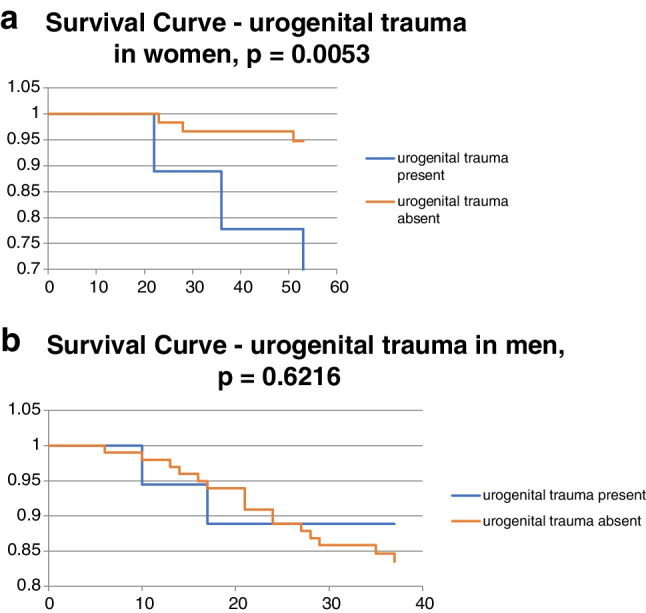
**a** Urogenital trauma in women. **b** Urogenital trauma in men

**Fig. 5:**
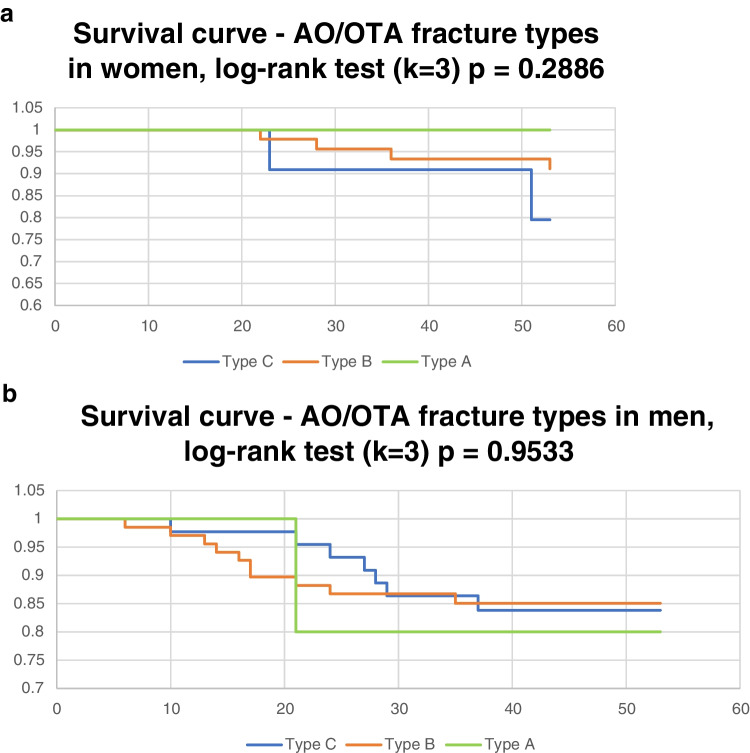
**a** AO/OTA fracture types in women. **b** AO/OTA types in men

**Fig. 6:**
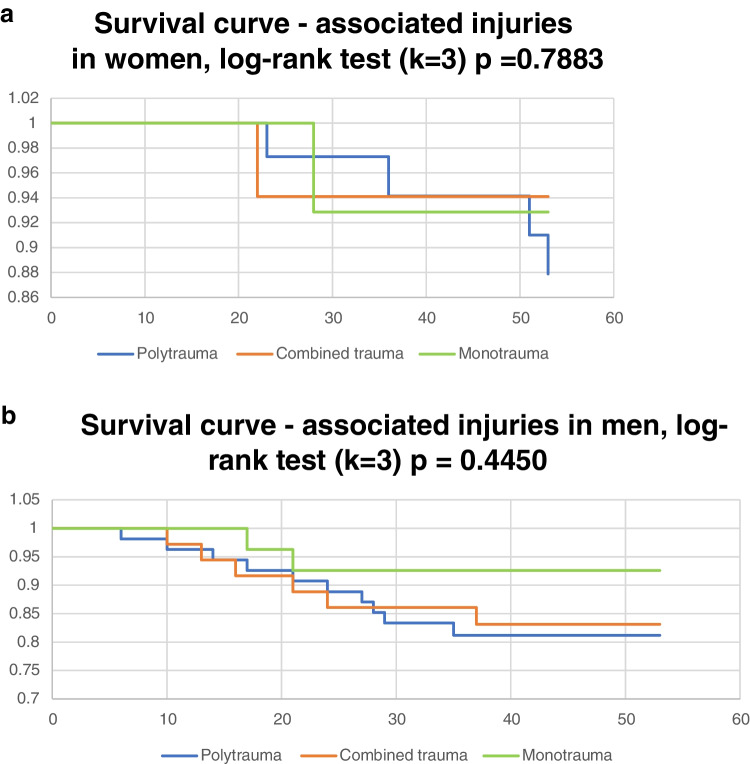
**a** Associated injuries in women. **b** Associated injuries in men

There were no significant independent risk factors for surgical site infections in the total cohort. However, as we noted disproportionality and heterogeneity between in the data for males and females, we divided the patients into two groups according to sex and evaluated the risk factors for these two groups individually. In the Cox regression model for the male group, no risk factors were significant (*p* = 0.5498). In the female group, however, the *p*-value for the Cox regression model was 0.0027. Among the female patients, age over 50 (risk ratio 11.19 (1.39–89.98), *p* = 0.0232) and coincidence of urogenital trauma (risk ratio = 6.56 (1.56–27.63), *p* = 0.0104) were identified as significant risk factors in 2 × 2 tables. The coexistence of both these risk factors further increased the risk of infectious complications requiring surgical revision. Further repeated Cox regression analyses were then performed in the female patients, using a select smaller numbers of risk factors. The smallest *p*-value was obtained by using age (above and below 50 years), urogenital trauma, and AO/OTA classification of pelvic fracture, which generated a *p*-value of 0.0006. The common risk ratio for age over 50 years and presence of urogenital trauma according to Cox regression was 212.59 (8.78–5148.68, *p* = 0.0010), which shows a significant interaction between the two risk factors (Tables 1 and 2). This relationship was confirmed using a 2 × 2 table (age above 50 years with urogenital trauma versus age under 50 without urogenital trauma). The Fisher’s exact test gave a *p*-value of 0.0003. The other risk factors for which no significance was demonstrated were processed in the form of Kaplan–Meier graphs with significance evaluation using a log-rank test (see Figs. 2, 3, 4, 5, and 6).

**Table 1:** Risk factors in the male and female patients

Male patients	Risk ratio	95% CI lower	95% CI upper	z	p-value	Fisher 2 × 2	Heterogeneity (male vs. female) Woolf test
Age	2.19	0.77	6.23	1.47	0.1428	0.1874	**0.0054**
Energy	0.75	0.20	2.80	− 0.43	0.6657	0.6507	0.9710
Urogenital trauma	0.69	0.17	2.74	− 0.53	0.5950	0.7355	**0.0288**
Fracture type							
C vs. B	1.08	0.44	2.63	0.17	0.8622	1.0000	0.4859
B vs. A	0.74	0.12	4.65	− 0.33	0.7438	0.5692	NS
C vs. A	0.80	0.12	5.21	− 0.24	0.8114	1.0000	NS
Associated trauma							
P vs. C	1.11	0.44	2.79	0.22	0.8224	1.0000	0.6787
C vs. M	2.25	0.49	10.29	1.05	0.2959	0.4483	0.6784
P vs. M	2.50	0.59	10.62	1.24	0.2143	0.3197	0.5077
Female patients	Risk ratio	95% CI lower	95% CI upper	z	p-value	Fisher 2 × 2	
Age	0.09	0.01	0.72	− 2.27	0.0232	0.0092	
Energy	0.76	0.10	5.80	− 0.26	0.7936	1.0000	
Urogenital trauma	6.56	1.56	27.63	2.56	0.0104	0.0270	
Fracture type							
C vs. B	2.09	0.44	10.00	0.92	0.3555	0.3264	
B vs. A	NS	NS	NS	NS	NS	0.5773	
C vs. A	NS	NS	NS	NS	NS	0.4762	
Associated trauma							
P vs. C	1.84	0.22	15.23	0.56	0.5727	1.0000	
C vs. M	0.82	0.06	12.01	− 0.14	0.8871	1.0000	
P vs. M	1.51	0.18	12.40	0.39	0.6993	1.0000	

*CI*, confidence interval; *P*, polytrauma; *C*, combined trauma; M, monotrauma

Boldfaced p values were significant

**Table 2:** Common risk ratio in female group (Cox regression model)

	*P*-value	exp(b)	95% CI	
			Lower	Upper
Age	**0.0319**	0.0914	0.0103	0.8128
Type	0.1085	6.5142	0.6608	64.2128
Urogenital trauma	**0.0099**	19.4328	2.0416	184.9656
Age and urogenital trauma	**0.0010**	212.59	8.78	5148.68
Age, urogenital trauma, fracture types C vs. B	**0.0037**	1384.828	10.54164	181921.2
Age, urogenital trauma, fracture types C vs. A	**0.0099**	9020.99	8.899774	9143858

*CI*, confidence interval

Boldfaced p values were significant

When comparing the risk ratio for individual factors, significant heterogeneity was identified between male and female patients in the case of age (*p* = 0.0054) and the presence of urogenital injury (*p* = 0.0288, Table 1).

In addition to qualitative analysis of age (above and below 50 years), age was also evaluated quantitatively as a parametric value in the Kruskal–Wallis test with post hoc comparison using paired Mann–Whitney-Wilcoxon tests in their exact form. Women with infections were significantly older than women without infections (*p* = 0.0105) and men with (*p* = 0.0118) and without infections (*p* = 0.0316). In contrast, there were no significant differences in the age of men with and without infection (*p* = 0.3697) (Tables 2 and 3).

**Table 3:** Mean age according to sex and AO/OTA fracture type

Sex and AO/OTA type	Mean	SD	95% CI lower	95% CI upper
W-A	36.73	16.90	26.74	46.71
M-I-C	38.86	18.89	24.86	52.85
W-B	40.45	14.32	36.12	44.78
M-C	40.49	17.30	34.91	46.06
M-I-B	42.00	12.19	34.44	49.56
W–C	44.22	24.57	28.17	60.27
M-B	47.71	15.26	43.78	51.64
M-A	48.75	24.90	24.34	73.16
M-I-A	55.00	NS	NS	NS
W-I-B	57.25	15.90	41.66	72.84
W-I-C	62.00	1.41	60.04	63.96
Significant post hoc pairwise comparison (Mann–Whitney–Wilcoxon exact tests)				
W-B	M-B	**0.0183**	Men with B type fractures were older than women with B type fractures	
W-I-C	M-I-B	**0.0303**	Women with infections and C type fractures were older than men with infections and B type fractures	
M-C	M-B	**0.0431**	Men with C type fractures were younger than men with B type fractures	
W-A	M-B	**0.0492**	Women with type A fractures were younger than men with type B fractures	
W-B	M-B	**0.0183**	Men with B type fractures were older than women with B type fractures	
W-I-C	M-I-B	**0.0303**	Women with infections and C type fractures were older than men with infections and B type fractures	

*W*, women; *M*, men; *I*, infection; *CI*, confidence interval; *SD*, standard deviation

Boldfaced p values were significant

**Fig. 7:**
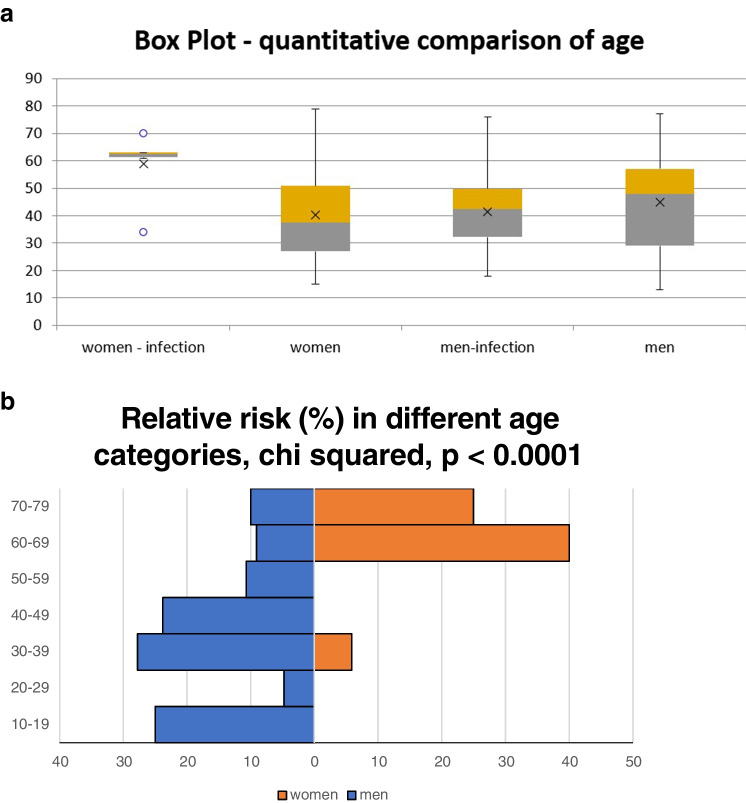
**a** Quantitative comparison of age. **b** Relative risk according in different age categories

The effect of age by sex and type of injury according to the AO/OTA classification was tested. Four comparisons were significant (for more details see Table 3). Women with infections and type C injuries were older than in men with infections and type B injuries. Comparison of the seven age categories (10-year age groups) by gender was highly significant (chi-squared 7 × 2, *p* < 0.0001). Paired comparisons are shown in the Fig. 7a and b, where it can be seen that women have the highest incidence of infections in the age group 60–69 (relative risk 40.0%), while men in the age category 30–39 (relative risk 27.78%), Fig. 7b. The basic AO/OTA types were not significant in men, and in Fig. 5a, an increased risk of infectious complications in women in type C can be seen (Fig. 5a). This observation was not apparent in men (Fig. 5b). There was not enough data for type A injuries to test the heterogeneity of multiple comparison models by type of injury in men and women.

**Fig. 8:**
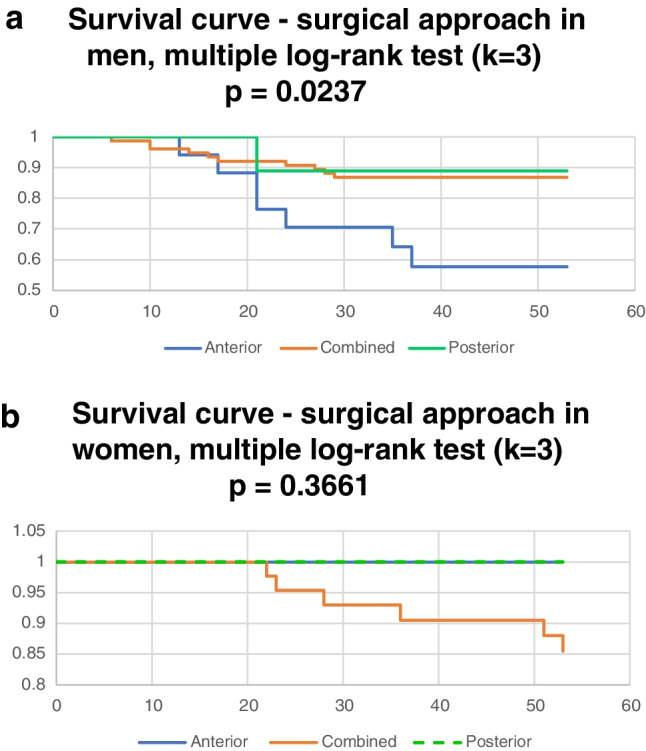
**a** Surgical approach in men. **b** Surgical approach in women

In men, the anterior approach proved to be a significant risk factor (in the Cox regression model for type of approach *p* = 0.0232), Fig. 8. The comparison of the anterior approach and combined approach was highly significant (risk ratio 3.13 (95% CI 1.39–7.04), *p* = 0.0058).

Furthermore, no significant effect of the osteosynthetic material used on the occurrence of serious infections was demonstrated in either group. The implants in patients undergoing re-operations consisted of 38 plates, four pubic screws, three iliac wing screws, 23 ilosacral screws, two transiliac internal fixators, four lumbopelvic fixations, and one other implant. Five patients were initially treated with temporary external fixation.

In women, infectious complications occurred only in cases where combined surgical approaches were used. However, when evaluating of the association of type of approach (anterior, posterior, or combined) with severe infectious, no significant differences were found (Cox regression for the approach *p* = 0.9247). Similarly, initial external fixation of the pelvic ring was not associated with a significantly higher risk of serious infectious complications, which may be due to the relatively lower number of patients with external fixation (33 patients in the whole group, 5 patients in the infection group, *p* = 0.7747). There was a relatively higher proportion of combined approaches and anterior plates with iliosacral screws in the group of patients with severe infections. Epidemiological data in these patients are shown in Table 4.

**Table 4:** (left columns) Epidemiologic data in patients undergoing reoperations for infections after pelvic osteosynthesis

Revision free interval (days)	Age	Sex	AO/OTA type	Associated trauma	High energy = 1, low energy = 0	Urogenital trauma	Time to surgery (days)	Follow-up (months)
28	18	M	C	Polytrauma	1	0	7	2
24	19	M	B	Combined trauma	0	0	6	19
21	25	M	C	Combined trauma	1	0	0	27
13	30	M	B	Combined trauma	1	0	0	8
29	32	M	C	Polytrauma	1	0	8	17
24	33	M	C	Polytrauma	1	0	2	2
10	35	M	B	Combined trauma	1	1	0	21
17	37	M	B	polytrauma	1	1	5	3
17	41	M	B	Monotrauma	1	0	5	14
27	44	M	C	Polytrauma	0	0	2	4
37	44	M	C	Combined trauma	1	0	13	30
6.0	46	M	B	Polytrauma	1	0	0	58
14	49	M	B	Polytrauma	1	0	1	60
35	50	M	B	Polytrauma	1	0	14	9
16	52	M	B	Combined trauma	1	0	3	5
21	55	M	A	Polytrauma	1	0	0	36
21	61	M	B	Monotrauma	1	0	6	5
10	76	M	C	Polytrauma	1	0	6	14
28	34	F	B	Monotrauma	1	0	7	36
23	61	F	C	Polytrauma	1	0	10	63
36	62	F	B	Polytrauma	1	1	0	5
51	63	F	C	Polytrauma	1	0	7	5
22	63	F	B	Combined trauma	0	1	4	1
53	70	F	B	Polytrauma	1	1	6	48

The primary surgical time did not differ significantly although this time was non-significantly longer in women with infection. The mean surgical time (± SD (95% CI)) was 90.0 ± 21.2 (60.6–119.4) min in men with infection vs. 107.6 ± 57.0 (89.5–125.8) without, *p* = 0.7410 and 161.7 ± 63.3 (90.0–233.3) min in women with infection vs. 99.5 ± 40.9 (82.0–117.0) with no infection; *p* = 0.0524.

The mean incision length in cm (sum of anterior and posterior incision) did not significantly differ regardless to sex (20.8 ± 6.7 (17.7–23.9) in men with infection vs. 22.7 ± 6.0 (21.4–24.0); *p* = 0.2809; 24.0 ± 6.0(19.2–28.8) in women with infection vs. 23.4 ± 5.6 (21.9–24.9); *p* = 0.6941).

Concerning the microbiological agents cultivated from the infected wounds, the most frequent finding was multiple pathogens, and the most common of which were coagulase-negative staphylococci, followed by Gram negative rods. The most frequently cultured pathogens are in Table 5.

**Table 5:** Etiological agents in patients undergoing reoperations for severe infections

Etiological agents	Antibiotics
coagulase-negative staphylococci; *Escherichia coli*	Oxacillin
coagulase-negative staphylococci	Cefuroxime
*Pseudomonas aeruginosa*	Meropenem, Cefazoline, Ampicillin, Amoxicilin—Clavulanate
coagulase-negative staphylococci	Clindamycin, Amoxicilin—Clavulanate
coagulase-negative staphylococci	Cefuroxim
coagulase-negative staphylococci; *Bacillus* spp.; *Enterococcus faecalis*	Cefuroxim
*Staphylococcus aureus;* coagulase-negative staphylococci; *Enterococcus faecalis; Pseudomonas aeruginosa; Proteus mirabilis; Bacteroides* spp.	Cefuroxim
coagulase-negative staphylococci; *Enterobacter* sp.; *Citrobacter* sp.	Cefuroxim
coagulase-negative staphylococci; *Enterobacter cloacae*	Clindamycin, Amoxicilin—Clavulanate
coagulase-negative staphylococci; *Enterococcus faecalis; Pseudomonas aeruginosa*	Cefuroxim
coagulase-negative staphylococci; *Pseudomonas aeruginosa*	Oxacillin
coagulase-negative staphylococci; *Bacillus* sp.	Oxacillin
coagulase-negative staphylococci; *Pseudomonas aeruginosa*	Amoxicilin—Clavulanate
*Escherichia coli; Serratia marcescens*; later *Enterococcus faecalis; Klebsiella pneumoniae*	Ceftazidine, Ciprofloxacine
coagulase-negative staphylococci	Amoxicilin—Clavulanate
coagulase-negative staphylococci	Oxacillin
*Staphylococcus aureus;* coagulase-negative staphylococci	Oxacillin
coagulase-negative staphylococci; *Escherichia coli*	Ampicillin-Sulbactam
*Enterococcus faecalis*	Cefuroxim
coagulase-negative staphylococci; *Klebsiella pneumoniae; Pseudomonas aeruginosa*	Oxacillin
coagulase-negative staphylococci; *Enterococcus faecalis; Escherichia coli; Pseudomonas aeruginosa*	Clindamycin
*Enterococcus faecalis; Enterobacter asburiae*	Amikacin
coagulase-negative staphylococci; *Escherichia coli*	Clindamycin, Oxacillin
coagulase-negative staphylococci; *Enterococcus faecalis; Enterobacter cloacae*	Cefuroxim

## Discussion

Soft tissue infections complicating the treatment of pelvic fractures can be classified according to location as occurring outside of the pelvic skeleton within the gluteal, pubic or inguinal regions, or as occurring inside the lesser and greater pelvis, where they may further be classified as extraperitoneal (pelvic pyomyositis) or intraperitoneal (pelveoperitonitis). Infections of the pelvic skeleton include exogenous osteomyelitis, septic sacroiliitis, secondary septic coxitis, and septic symphysitis [3, 7]. One than one of these types of infection may occur simultaneously, and in such, cases it is not possible to pinpoint the primary source of infection (e.g., concomitant pelvic pyomyositis and septic sacroiliitis). Thus, it is sometimes better to classify pelvic infections according to the depth of the infected tissues into superficial and deep infections, depending on whether or not they penetrate the gluteal fascia, superficial abdominal fascia, or fascia lata [7]. Wise et al. have recently described these criteria. A deep surgical site infection according to the CDC involves the deep soft tissues of the incision and presents with at least one of the following: purulent drainage from the deep incision; a deep incision that spontaneously dehisces or is opened by the surgeon when the patient has clinical symptoms of infection including fever, localized pain and/or tenderness, or an abscess; or other evidence of deep infection is found on direct examination, during re-operation, or by histopathologic or radiologic examination [28]. According to the need for conservative or surgical treatment, pelvic surgical site infections can be classified as mild infections, which can be managed by conservative treatment and severe infections, which require surgical treatment.

For diagnosing surgical site infection after the operative treatment of pelvic fractures, we rely on local signs of infections, the qSOFA score, laboratory signs (leukocytosis with left shift and elevation of CRP and procalcitonin), and imaging method findings (sonography of the soft tissues in the region of the surgical wound and computed tomography of the pelvis). Similar diagnostic methods have been recommended by many other authors [3, 7, 10, 11, 16, 28]. Additionally, other authors recommend using a combination of SPECT and CT or SPECT and MRI, as this has a higher sensitivity for diagnosing infections. In particular, MRI has an especially high sensitivity for subacute and chronic infections [16]. When SPECT is not available PET/CT can be used, but it is not as sensitive for skeletal infections [16]. Antibiotic therapy is ideally started after culturing microbiological swabs from the surgical wound, secretion samples, or tissue samples taken at surgical revision [17, 28]. When patients present with febrility or positive qSOFA assessments, blood cultures are taken and further examinations are then performed to find the focus of the infections (such as urine and nasopharyngeal cultures) [16, 17, 28].

Conservative treatment for mild infections consists of regular wound dressing changes with irrigation with antiseptic solutions, local antibiotics, removal of sutures, and excision of devitalized tissue in local anesthesia [7, 16, 17]. Surgical treatment depends on instability of the pelvic ring, the length of time from the injury and the extent of the infection. Soft tissue infections without concomitant pelvic instability can be treated by surgical revision with excision of the inflamed and devitalized tissue and lavage and drainage [13]. When a skin defect results after such treatment, it can be treated by using vacuum assisted closure or closed with a skin graft with the aid of a plastic surgeon [13, 14, 16]. Cases of osteomyelitis and failure of osteosynthesis should be treated by removing osteosynthetic material, stabilization of the pelvic ring by external fixation, and in cases of instability of the posterior segment by mini-invasive fixation (for example, iliosacral screws) [4]. In cases of late onset infections that occur after complete healing, the osteosynthetic material should be removed, and the operative field should be irrigated and drained [7].

Cases of infected non-union are a separate issue. In the anterior segment, in addition to decortication, resection of the non-union and vascularized bone grafts can be used. This is particularly useful for cases of septic destruction of the symphysis. Vascularized fibular autografts are a good option, but require cooperative with a plastic surgeon [13, 14, 16]. Infected non-union of the posterior segment is a more problematic topic and requires complex treatment. For infected non-union of the posterior pelvic ring, it is necessary to use a combined anterior and posterior approach, consisting of, for example, a posterior revision, followed by an anterior revision, and then finished via the posterior approach [13, 14, 16]. In addition to revision and resection of infected surfaces of the non-unions, posterior decompression of the sacral roots can also be performed followed by bone grafting, which can be combined with local antibiotics. Local antibiotics can be applied using calcium sulphate beans, an example of which is Stimulan® [17]. Bridging posterior stabilization is performed either by individual long iliosacral screws with threads along the entire length, in some cases in combination with transiliac internal fixation, or spinopelvic fixation. This type of fixation cannot be used if there are simultaneous soft tissue defects in the posterior segment. Alternatively, 3D-navigated percutaneous sacral rods, implanted into the body of vertebra S1 or even to S2 can be used [1, 2, 4, 5].

The optimal length of antibiotic treatment for pelvic infections is a controversial topic. In our centre for bone and joint infections or secondary pelvic pyomyositis, we give intravenous antibiotics for three weeks and then peroral antibiotics for another three weeks. For isolated soft tissue infections, we give intravenous antibiotics for two weeks, followed by peroral antibiotics for another two weeks. Each case is individualized according to the results of the cultivation, and each case is consulted with a microbiologist (see also Table 5).

Antibiotics are also given locally using various carriers such as beads, sponges, and bone cement. Antibiotics can be given in cement-augmented implants, which, although limited in clinical practice by their high price, are especially useful in patients with osteoporosis. Types of augmented implants used in pelvic fractures include augmented iliosacral screws, augmented spinopelvic fixators, and augmented transiliac internal fixators [17]. The treatment strategy for infectious complications after pelvic ring stabilization requires an individual approach and good interdisciplinary cooperation as guidelines based on prospective clinical trials are still lacking.

In terms of cohort sizes, our study is comparable with two recent studies also devoted to pelvic infections: Bakhshayesh et al. 2018 [2] and Kanakaris et al. 2021 [10]. Bakhshayesh et al. reported a similar proportion of reoperations for surgical site infections as in our study. Also, similarly to our study, no risk factors were identified for the cohort as a whole. However, in contrast with our study, a post hoc analysis on sex-based subgroups was not performed [2]. In Kanakaris et al.’s study, a larger number of risk factors were investigated, logistic regression was used for the analysis rather than Cox regression, and in their multivariate analysis ISS score, diabetes, a posterior approach, and alcohol abuse were all identified as independent risk factors [10]. Other studies focused on fewer risk factors than our study. Jaeblon et al. [8] monitored the effect of obesity on the incidence of infectious complications and showed that a waist hip ratio is a better predictor of infectious complications than body mass index. A waist-hip ratio above 1.0 was identified as a strong risk factor as is an indicator of significant abdominal obesity [8, 18]. Waist-hip ratio was not a monitored parameter in our retrospective study.

**Fig. 9:**
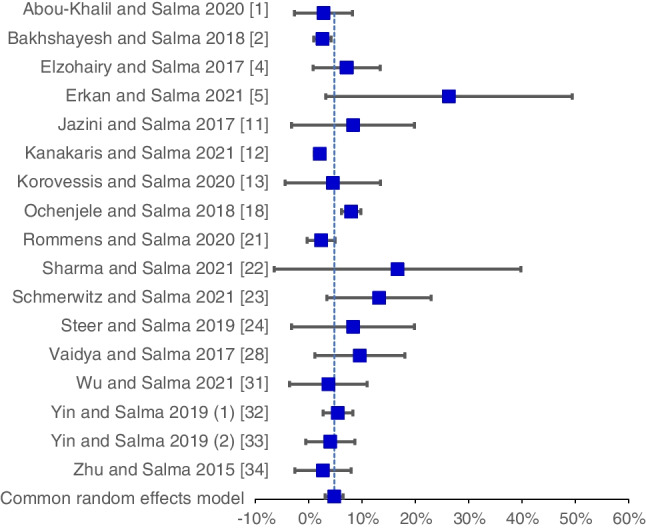
Meta-analysis of recent studies

The influence of concomitant urogenital trauma has not only been investigated by orthopedic studies, but also by urological and gynecological studies. Most of these studies are case reports, exceptions being Testa et al.’s literature review [25] and Li et al.’s case series [12], investigating the relationship between pelvic fractures and vaginal injuries. There are also several epidemiological studies, with study populations much larger than ours, to which we can only make indirect comparison [6]. By contrast, most studies investigating pelvic ring stabilization focus on one to two types of implants and are smaller than our study [7, 9, 11, 14]. To create an overall estimate of the incidence of severe pelvic infections after surgical stabilization, we performed a metanalytic comparison of case series from the literature. The results are shown in Table 6 and Fig. 9. As significant heterogeneity was found between the studies, we decided to use a random effects model. The pooled infection rate was 4.8% (95% CI 3.1–6.5%). Although the incidence of infections in our study was relatively higher, this may be due to the inclusion of all patients regardless of the procedure they underwent. It was well known, for example, that patients undergoing open reduction and internal fixation have a higher risk of infectious complications than patients undergoing mini-invasive stabilization.

**Table 6:** Comparison of infection rates in recent studies

Study	Infection	Total	Rate	95% CI lower	Upper
Abou-Khalil et al. 2020 [1]	1	36	0.0278	− 0.0267	0.0822
Bakhshayesh et al. 2018 [2]	10	385	0.0260	0.0099	0.0421
Elzohairy and Salama 2017 [4]	5	70	0.0714	0.0088	0.1340
Erkan et al. 2021 [5]	5	19	0.2632	0.0325	0.4938
Jazini et al. 2017 [9]	2	24	0.0833	− 0.0322	0.1988
Kanakaris et al. 2021 [10]	18	858	0.0210	0.0113	0.0307
Korovessis et al. 2020 [11]	1	22	0.0455	− 0.0436	0.1345
Ochenjele et al. 2018 [16]	73	913	0.0800	0.0616	0.0983
Rommens et al. 2020 [19]	3	128	0.0234	− 0.0031	0.0500
Sharma et al. 2021 [19]	2	12	0.1667	− 0.0643	0.3977
Schmerwitz et al. 2021 [20]	7	53	0.1321	0.0342	0.2299
Steer et al. 2019 [22]	2	24	0.0833	− 0.0322	0.1988
Vaidya et al. 2017 [26]	5	52	0.0962	0.0119	0.1804
Wu et al. 2021 [29]	1	27	0.0370	− 0.0356	0.1096
Yin et al. 2019 (1) [30]	15	272	0.0551	0.0272	0.0831
Yin et al. 2019 (2) [31]	3	74	0.0405	− 0.0053	0.0864
Zhu et al. 2015 [32]	1	37	0.0270	− 0.0259	0.0800
Total—fixed effects model			0.0350	0.0283	0.0417
Total—random effects model			0.0480	0.0310	0.0650

Heterogeneity: *Q* = 48.78, d.f. = 16, *p* < 0.0001, *I*^2^ = 67.20%

Our study is limited by its retrospective monocentric design. Another limitation is that an analysis of long-term radiological and functional outcomes is missing.

Omitting of evaluation of initial lab test (e.g., initial hemoglobin, total proteinemia, and level of albumin) is also gross limitation of our study, but we did not have sufficient data set to assessed covariates such as significant comorbidities (e.g., chronic liver disease, chronic renal dysfunction, chronic obstructive pulmonary disease, diabetes, chronic heart failure), age and sex that all could be associated with change in haemoglobin and albumin regardless of primary pelvic ring injury. Both severe initial anaemia and hypoproteinaemia might be coupled with a poorer functional outcome and with a significantly higher risk of infection. Routine vitamin D level screening was also missing in our study, sarcopenia and muscle weakness cloud be worsened by severe depletion of vitamin D, especially in elderlies. We screened only patient up 65 years—in case of hypovitaminosis, we addressed patients for peroral supplementation.

On the other hand, the strengths of the study are its relatively large patient cohort, the inclusion of patients with all types of implants and surgical approach, the graphical evaluation of the results, and the analysis of the annual number of surgical procedures performed for pelvic ring injuries and severe infections requiring reoperations.

## Conclusion

A relatively high number of serious infectious complications occurred in our cohort of patients who underwent surgery for fractures of the pelvis. Evaluation of the results showed that in female patients being over 50 years of age and having concomitant urogenital trauma were independent risk factors for surgical site infections. The combined effect of having both these risk factors substantially increased the risk of infectious complications. Women in the 60–69 age group and men aged 30–39 were most at risk of infection. Even through type B and C pelvic injuries and cases polytrauma were more frequently associated with infectious complications, this finding did not reach statistical significance. Similarly, the energy of the trauma was also insignificant. Infectious complications occurring in men were more frequently associated with an open approach, while in women they were more frequently associated with a combined approach. Only prospective studies, ideally with multicenter cooperation will be able to definitively elucidate the risk factors for infectious complications after pelvic osteosynthesis and validate of our results. For the diagnosis and treatment of infectious complications after pelvic osteosynthesis, we strongly recommend a multidisciplinary and individualized approach.

## Data Availability

Complete excel electronic data sets are available at authors on request.
